# Prognostic Influence of Galectin-1 in Gastric Adenocarcinoma

**DOI:** 10.3390/biomedicines12071508

**Published:** 2024-07-07

**Authors:** Cristina Díaz del Arco, Lourdes Estrada Muñoz, María de los Ángeles Cerón Nieto, Elena Molina Roldán, María Jesús Fernández Aceñero, Soledad García Gómez de las Heras

**Affiliations:** 1 Department of Legal Medicine, Psychiatry and Pathology, School of Medicine, Complutense University of Madrid, 28040 Madrid, Spain; 2 Department of Pathology, Hospital Clínico San Carlos, Health Research Institute of the Hospital Clínico San Carlos (IdISSC), 28040 Madrid, Spain; m.delosangeles.ceron@salud.madrid.org; 3 Department of Pathology, Rey Juan Carlos Hospital, 28933 Móstoles, Spain; lourdes.estrada@hospitalreyjuancarlos.es; 4 Department of Basic Medical Sciences, School of Medicine, Rey Juan Carlos University, 28933 Móstoles, Spain; soledad.garcia@urjc.es; 5 Biobank, Hospital Clínico San Carlos, 28040 Madrid, Spain; elenamilagrosa.molina@salud.madrid.org

**Keywords:** galectin-1, gastric cancer, prognosis, stromal, epithelial, immunohistochemistry

## Abstract

Galectin-1 (Gal-1), a member of the human lectin family, has garnered attention for its association with aggressive behavior in human tumors, prompting research into the development of targeted drugs. This study aims to assess the staining pattern and prognostic significance of Gal-1 immunohistochemical expression in a homogeneous cohort of Western patients with gastric cancer (GC). A total of 149 cases were included and tissue microarrays were constructed. Stromal Gal-1 expression was observed to some extent in most tumors, displaying a cytoplasmic pattern. Cases with stromal Gal-1 overexpression showed significantly more necrosis, lymphovascular invasion, advanced pTNM stages, recurrences, and cancer-related deaths. Epithelial Gal-1 expression was present in 63.8% of the cases, primarily exhibiting a cytoplasmic pattern, and its overexpression was significantly associated with lymphovascular invasion, peritumoral lymphocytic infiltration, and tumor-related death. Kaplan/Meier curves for cancer-specific survival (CSS) revealed a significantly worse prognosis for patients with tumors exhibiting stromal or epithelial Gal-1 overexpression. Furthermore, stromal Gal-1 expression stratified stage III patients into distinct prognostic subgroups. In a multivariable analysis, increased stromal Gal-1 expression emerged as an independent prognostic factor for CSS. These findings underscore the prognostic relevance of Gal-1 and suggest its potential as a target for drug development in Western patients with GC.

## 1. Introduction

Gastric cancer (GC) ranks as the fifth most common malignant neoplasm and the third leading cause of cancer-related mortality [1]. The incidence of GC exhibits substantial regional disparities, with high-risk zones predominantly observed in East Asia and comparatively lower risk in many Western nations [2]. While certain histological subtypes of GC are influenced by genomic abnormalities, rendering them less amenable to preventive measures, others exhibit clear associations with environmental factors, presenting opportunities for prevention [3,4]. Despite advancements in targeted therapies following the identification of HER-2 amplification and the development of antiangiogenics and immunotherapy in GC, the mortality rate still remains high [5]. This is mainly attributed to late-stage diagnoses, which impede the feasibility of curative surgical resection [6]. Additionally, the significant heterogeneity at both the phenotypic and molecular levels of this tumor may be hindering the discovery of novel biomarkers and the stratification of patients for personalized management [7]. Indeed, limited progress has been made in targeted therapies following the discovery of the aforementioned treatments. However, the development of anti-claudin therapies has opened a horizon of hope for advanced GC patients, and numerous ongoing trials are exploring novel and promising targets such as FGFR and MET, new antiangiogenic, immunotherapeutic, and anti-HER2 approaches, as well as innovative drug delivery systems [8,9,10,11,12].

### 1.1. Structural Characteristics of Galectins

**Figure 1:**
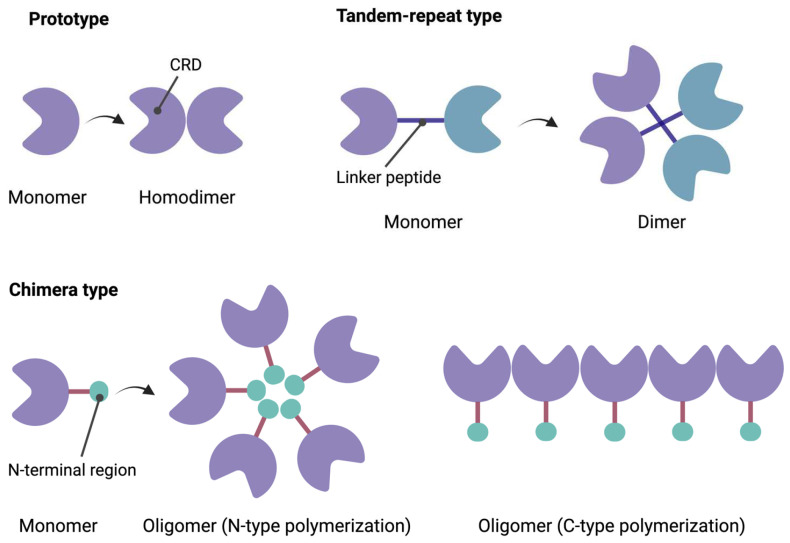
Structure of galectins. CRD: carbohydrate recognition domain. Created with BioRender.com under an individual license (Dr. Díaz del Arco).

Human lectins comprise a diverse group of proteins characterized by their specific interactions with carbohydrates [13]. These molecules are classified into five groups according to the structure of their specific carbohydrate-recognizing domain (CRD). The type S lectins, also known as galectins, show affinity for β-galactosides [13,14]. Fifteen subtypes of galectins have been identified, including galectin-1 (Gal-1) [15]. The galectin family can be classified into three groups: prototypic single-CRD galectins, which can form non-covalent homodimers (Gal-1, 2, 5, 7, 10, 11, 13, 14, and 15); galectins with tandem repeats of two similar CRD motifs (Gal-4, 6, 8, 9, and 12); and the chimera-type, to which Gal-3 belongs, containing a single CRD and capable of oligomerization (Figure 1) [16,17]. Gal-1 is a prototypic galectin composed of 135 amino acids and two identical CRDs, capable of existing in monomeric or homodimeric forms [18]. It has a three-dimensional beta-sandwich structure, with two opposing antiparallel beta-sheets [19,20].

### 1.2. Functional Roles of Galectin-1

**Figure 2:**
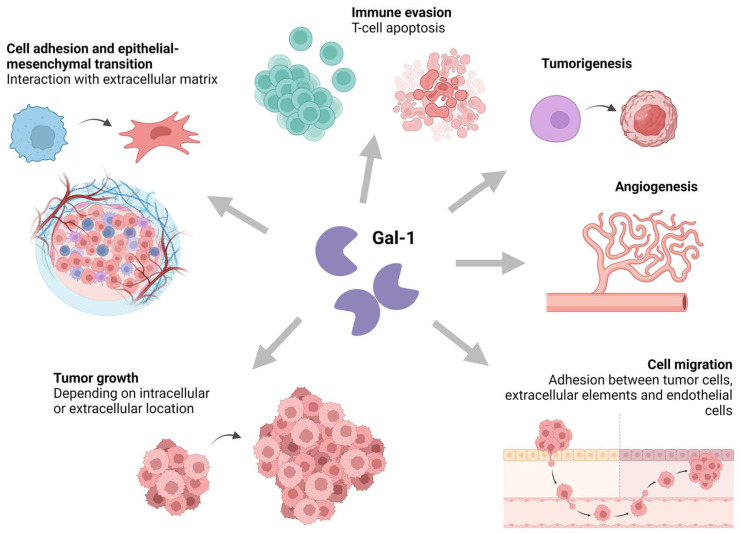
Key roles of Gal-1 in tumor development and metastases. Created with BioRender.com under an individual license (Dr. Díaz del Arco).

Gal-1 exhibits a dual localization, being present intracellularly as well as extracellularly, with its secreted form implicated in various cellular processes such as cell adhesion, migration, proliferation, and survival, alongside proangiogenic and immunosuppressive effects (Figure 2) [21]. Notably, Gal-1 has been associated with H-RAS activation and downstream signaling pathways [22]. In vitro studies have demonstrated that the inhibition of Gal-1 reduces tumor growth, with corroborating evidence across diverse human neoplasms including cervical, breast, lung, or head and neck cancers [23]. Furthermore, Gal-1 promotes tumor metastasis by modulating adhesion molecules in the tumor stroma and interacting with immune-related pathways [24,25,26]. The molecular mechanisms underlying galectin actions are still being investigated due to their complexity and diversity. Galectins are implicated in multiple cancer-related signaling pathways beyond H-RAS, including tyrosine kinase receptor pathways, the PD-1/PD-L1 axis, various apoptotic pathways, the JAK/STAT pathway, the NF-κB pathway, or pathways involved in cell cycle regulation [27,28,29,30,31].

To date, several studies have examined the expression of Gal-1 in GC and its potential prognostic significance. However, the majority of these studies have been conducted in Asian populations, making it necessary to investigate this molecule in Western cases given the distinct clinical, histological, molecular, prognostic, and treatment characteristics of these patients [32]. Our objective in this study was to assess the immunohistochemical (IHC) expression of Gal-1 in both stroma and epithelium, and to explore its correlation with clinicopathological factors and the prognosis of a series of Western patients who underwent surgical resection for GC.

## 2. Materials and Methods

This retrospective cohort study examined patients who underwent surgical resection for GC with curative intent at a tertiary institution (Hospital Clínico San Carlos, Madrid, Spain). The patients were identified through a comprehensive search of the Surgical Pathology Department’s database (Pat-Win) spanning from 2001 to 2014. Inclusion criteria encompassed the patients who underwent oncologic gastric resection with clear margins and D2 lymphadenectomy, excluding those who received neoadjuvant therapy and with distant metastases at diagnosis. Only the cases with well-preserved formalin-fixed paraffin-embedded tumor specimens were included in the final IHC analysis. Demographic, clinical, endoscopic, and radiological data were retrieved from electronic hospital records. Histopathological slides were reviewed by two independent pathologists, assessing microscopic variables including histologic type according to the Laurén and WHO classifications, tumor grade, growth pattern (expansive vs. infiltrative), the presence of signet ring cells, lymphovascular invasion, perineural invasion, tumor necrosis, intratumoral and peritumoral lymphocytic infiltration, desmoplasia, and budding. Tumor grade was determined based on the percentage of gland formation (low grade if glandular structures comprised 50% or more of the tumor). Tumor budding was evaluated according to the methods outlined by Ueno et al. [33,34]. The tumors were staged according to the 8th edition of the American Joint Committee on Cancer staging manual [35]. The lymph node ratio (LNR) was calculated as the ratio of metastatic lymph nodes to the total number of retrieved lymph nodes.

The primary outcome measure of the study was cancer-specific survival (CSS), defined as the time interval between surgery and tumor-related death, measured in months.

### 2.1. Immunohistochemical Study

Tissue microarrays (TMAs) were constructed from tumor tissue blocks, with each TMA containing two cores per patient representing both the tumor center and the leading edge. The MTA-1 tissue arrayer (Beecher Instruments, Sun Prairie, WI, USA) was employed for this purpose. Each core, measuring 1 mm in diameter, was meticulously punched from pre-selected tumor regions within the formalin-fixed paraffin-embedded blocks. IHC targeting Gal-1 was performed on 4-micron sections from the TMAs using a commercially available antibody (ab112525, Abcam, Cambridge, UK) diluted at a ratio of 1/50. The slides were independently reviewed by two pathologists, Estrada Muñoz L. and Fernández Aceñero MJ., who were blinded to the patient prognosis. Scoring was conducted using the H score, which integrates both the percentage of positivity and staining intensity, calculated separately for both the epithelial and stromal compartments. Intensity was assessed on a scale of 1–3 (mild, moderate, and intense), and the percentage of the stained cells ranged from 1–100%. Consequently, the staining values could range from 0 to 300.

### 2.2. Statistical Analysis

All the data were anonymized and stored in an Excel file for analysis using the SPSS 27.0 for Windows statistical package. Associations between the variables were assessed using either the χ^2^ chi-squared test (for qualitative variables) or Student’s *t*-test (for comparing means between dichotomous quantitative Gaussian variables). Non-parametric tests, such as Mann/Whitney U, were used for quantitative variables that did not have a Gaussian distribution according to the Kolmogorov/Smirnov test. Statistical significance was set at a *p*-value < 0.05. Survival curves were generated using the Kaplan/Meier method, with significance tested via the log-rank test. Multivariable Cox regression models were adjusted for potential confounders.

### 2.3. Ethical Approval

This study was approved by the drug research ethics committee of Hospital Clínico San Carlos (CEIm Hospital Clínico San Carlos, approval code: C.I. 16/017-E), with a subsequent amendment in June 2021.

## 3. Results

### 3.1. Clinicopathological Characteristics of the Study Cohort

The main features of our cohort are outlined in Table 1. The mean age was 72 years, with the majority of the patients presenting symptoms at diagnosis (89.3%). Most tumors were located in the gastric antrum (56.2%) and exhibited a fungoid (38.7%) or ulcerative (31.7%) morphology. The tumors were predominantly of the intestinal type (59.2%), followed by diffuse (29.3%) and mixed (11.6%) types. Necrosis, signet ring cells, lymphovascular invasion, and perineural infiltration were observed in 27%, 42.9%, 43.2%, and 45.3% of the cases, respectively. Intense intratumoral and peritumoral lymphocytic infiltration were identified in 76.1% and 27.4% of the cases, respectively. Most patients were diagnosed with pT3 tumors (61.6%) and presented with lymph node metastases (67.6%). The primary surgical approach was subtotal gastrectomy (70.9%) with D2 lymphadenectomy. In 61.3% of the cases, 16 or more lymph nodes were resected. Adjuvant therapy was administered in 23.2% of the patients. During follow-up, 44.1% of the tumors recurred, and 26.6% of the patients died due to GC.

### 3.2. Gal-1 Immunohistochemical Expression

**Figure 3:**
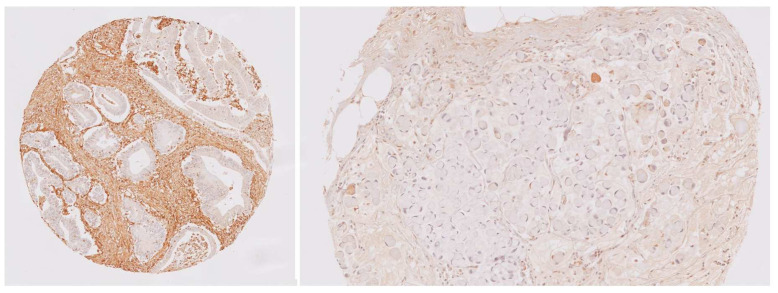
Examples of Gal-1 staining. Left: moderate to intense positivity in the stroma. Gal-1 IHC, ×100. Right: mild positivity in the stroma. Gal-1 IHC, ×200.

The characteristics of Gal-1 IHC expression are summarized in Table 2. Overall, 96% of the cases showed some degree of expression in stromal cells, with a cytoplasmic staining pattern observed in all these cases. Mild and moderate staining were observed in 72.6% and 27.4% of the cases, respectively (Figure 3). Regarding epithelial Gal-1 expression, 63.8% of the cases showed some degree of staining in the tumor cells. Of these, 86.3% exhibited cytoplasmic staining, with the majority showing mild intensity (72.6%).

The cutoff values for positive staining were established using the ROC curve analysis, with the epithelial Gal-1 scores ≥ 22.50 and stromal Gal-1 scores ≥ 27.50 considered positive. According to this classification, positive Gal-1 expression in tumor stroma and epithelial cells was identified in 32.2% and 42.3% of the cases, respectively.

### 3.3. Association between Gal-1 Expression and Clinicopathological Findings

The association between the stromal and epithelial Gal-1 expression and clinicopathological parameters is summarized in Table 3 and Table 4, respectively.

In univariable analysis, increased stromal Gal-1 expression, based on the established cutoff point, was associated with tumor necrosis, lymphovascular invasion, pTNM stage, recurrence, and cancer-related death (Table 3). There was also a trend towards significance in the relationship between the stromal Gal-1 expression and peritumoral lymphocytic infiltration, presence of lymph node metastases, and pN stage (*p* < 0.07).

On the other hand, the patients with tumors positive for epithelial Gal-1, according to our cutoff, exhibited significantly more lymphovascular invasion, peritumoral lymphocytic infiltration, and cancer-related deaths (Table 4). The relationship between epithelial Gal-1 expression, tumor grade, and pTNM stage showed a trend towards significance.

### 3.4. Association between Cancer-Related Death and Clinicopathological Factors

#### 3.4.1. Univariable Analysis

Univariable analysis revealed that cancer-related death was associated with tumor necrosis, diffuse-type tumors, lymph node involvement (pN stage, LNR, and median number of metastatic lymph nodes), age at diagnosis, and both the positive stromal and epithelial expression of Gal-1 (Table 5). The presence of signet ring cells showed a trend towards significance (*p* = 0.051).

#### 3.4.2. Kaplan/Meier Curves

**Figure 4:**
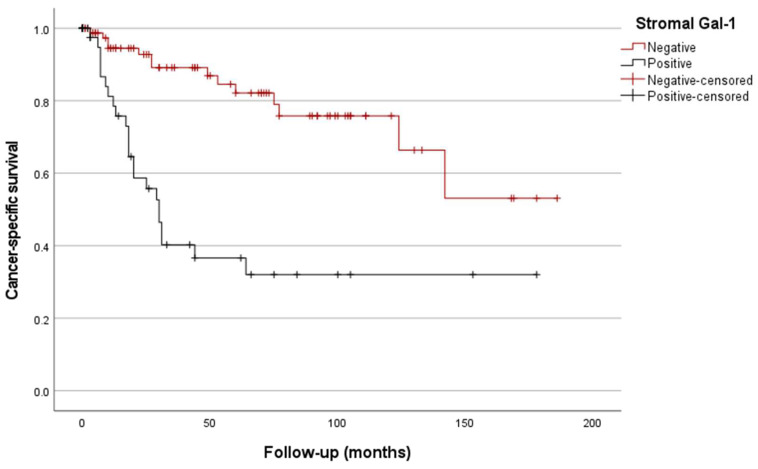
Kaplan/Meier survival curves depending on stromal Gal-1 IHC expression (*p* < 0.001).

**Figure 5:**
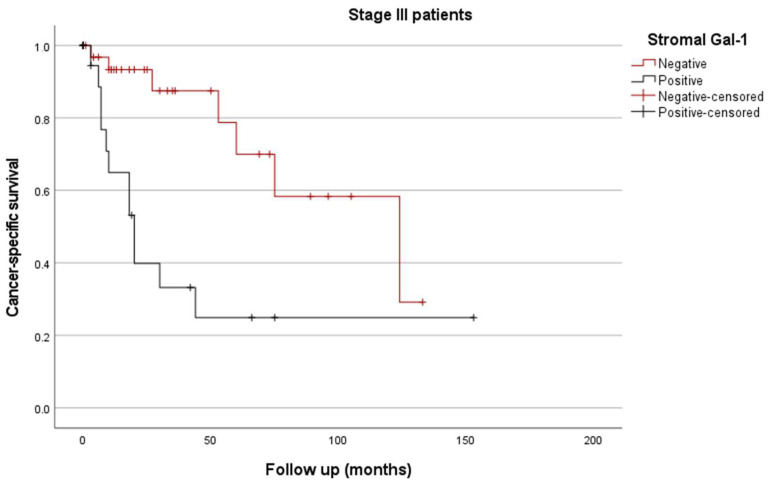
Kaplan/Meier survival curves of stage III gastric carcinoma depending on stromal Gal-1 IHC expression (*p* = 0.002).

Kaplan/Meier curves for CSS demonstrated that increased stromal Gal-1 expression was associated with significantly worse CSS compared to the patients without Gal-1 overexpression (estimated mean CSS of 139 vs. 72 months, respectively; *p* < 0.001, Figure 4). Furthermore, positive stromal Gal-1 expression stratified the stage III patients into two subgroups with significant prognostic differences (estimated mean CSS of 97 vs. 50 months; *p* = 0.002, Figure 5).

**Figure 6:**
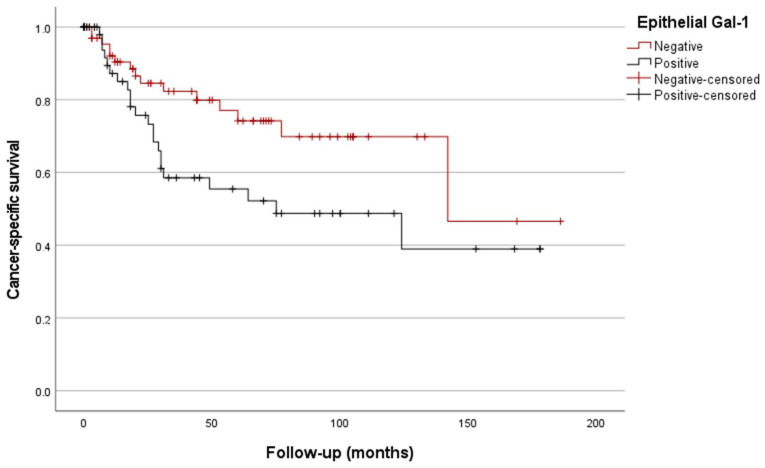
Kaplan/Meier survival curves depending on epithelial Gal-1 IHC expression (*p* = 0.037).

Similarly, the classification of the patients based on the epithelial Gal-1 expression also showed significant prognostic differences (estimated mean CSS of 129 vs. 95 months; *p* = 0.037, Figure 6).

#### 3.4.3. Multivariable Analysis

The results of the Cox regression analysis for CSS are presented in Table 6. Independent prognostic factors included diffuse Lauren type, LNR, and increased stromal Gal-1 expression.

## 4. Discussion

As previously mentioned, Gal-1 belongs to the lectin superfamily, specifically the prototypical group, characterized by its high-affinity binding to β-galactosides through a single CRD [36,37,38]. In tumorigenesis, Gal-1 may play roles in cancer growth, the development of metastasis, and immune evasion [39]. Intracellularly, Gal-1 drives tumorigenesis via the RAS/RAF pathway and increases H-RAS nanoclusters [40]. The increase in extracellular Gal-1 concentration correlates with tumor aggressiveness, the acquisition of a metastatic phenotype, tumor angiogenesis, and immune evasion [41]. In GC, Gal-1 promotes vasculogenic mimicry (VM) by activating the epithelial-mesenchymal transition (EMT) pathway, providing essential nutrients for tumor growth [42,43,44]. Additionally, the connections between blood vessels and VM in tumor tissues facilitate the direct access of tumor cells to the bloodstream [43,45]. In the context of EMT in GC, Gal-1 also activates the TGF-β/Smad signaling pathway, induces the expression of Gli-1 through a non-canonical hedgehog pathway, and promotes the expression of sphingosine-1 phosphate receptor-1 (S1PR1) [46].

The aberrant expression of Gal-1 has been described in various tumor tissues, including GC, ovarian carcinoma, hepatocellular carcinoma (HCC), colon carcinoma, renal cell carcinoma, breast carcinoma, cholangiocarcinoma, squamous cell carcinoma of the head and neck, lung carcinoma, urothelial carcinoma, prostate carcinoma, Kaposi’s sarcoma, Hodgkin lymphoma, and glioblastoma multiforme, often associated with poor survival [47,48,49,50,51,52,53,54,55,56,57,58,59,60,61,62,63,64,65,66,67,68]. Three meta-analyses exploring the prognostic influence of Gal-1 expression in different cancers have been published [41,69,70].

In our study, we observed a high prevalence of the stromal expression of Gal-1 in GC, with a predominant cytoplasmic localization. Specifically, moderate stromal staining was observed in 41.6% of our cases. Additionally, epithelial staining was found in 63.7% of the cases, also predominantly displaying a cytoplasmic pattern, with 27.4% of the cases showing moderate staining. Gal-1 overexpression was detected in the stroma and epithelium of GC in 32.2% and 42.3% of the cases, respectively, based on our established cutoffs. Previous research on GC has primarily identified Gal-1 overexpression in the tumor-associated stroma [47,59,64,65,66,67]. Interestingly, in some studies, Gal-1 overexpression was not detected in tumor cells. For instance, Bektas et al. (2010) reported weak Gal-1 expression in tumor epithelium in 15.1% of the cases and moderate expression in 3.2%, with no intense expression observed in tumor cells [63]. They noted Gal-1 expression in stromal cells in most tumors (>98%), distributed as mild (22.8%), moderate (28.3%), or intense (48.9%). Similarly, Zheng et al. (2016) observed intense Gal-1 expression in tumor-associated stroma but weak or negative expression in tumor cells [66]. In 2014, He et al. found that Gal-1 overexpression in cancer-associated fibroblasts (CAFs) enhanced GC cell migration and invasion in vitro [47]. They reported 94 cases of GC with Gal-1 overexpression in CAFs observed in 60.6% of them, while tumor cells showed negative staining. In contrast, Chong et al. in 2016 identified significantly more Gal-1 expression in tumor cells and stroma in GC cases compared to matched non-cancerous tissue samples [67].

Despite these discrepancies, the Gal-1 expression in both tumor epithelium and stroma is logical in light of previous studies. Tumor cells expressing Gal-1 have been shown to synthesize and secrete Gal-1 in stromal cells, and vice versa, as they are stimulated by the same tumor cells [71]. On the other hand, the higher expression of Gal-1 in tumor stroma is consistent with the described involvement of this molecule in the tumor microenvironment, affecting the extracellular matrix and various stromal cell types such as mesenchymal stem cells, macrophages, inflammatory cells, and fibroblasts through various mechanisms [47,48,59,67].

Regarding patient prognosis and consistent with the previous literature, our study observed a significant difference in terms of CSS for the patients with Gal-1 overexpression in tumor stroma and epithelium. However, in multivariable analysis, only stromal Gal-1 was identified as an independent prognosticator, along with Laurén’s diffuse type and LNR.

Several studies have analyzed the prognostic value of Gal-1 expression in GC, both in tumor epithelium and tumor-associated stroma [47,59,64,65,66,67]. In 2018, Wu et al. conducted a meta-analysis comprising 18 studies involving 2674 patients with various malignancies, including six articles focusing on digestive tumors (three GC and three HCC) [41]. They reported that the overexpression of Gal-1 was associated with lower OS (HR: 1.79, 95% CI: 1.54–2.08, *p* < 0.001), suggesting its potential as a prognostic factor in malignant tumors, particularly in digestive cancers. In 2019, Huang et al. obtained similar results in their meta-analysis of 29 studies involving 3543 cases of 13 different cancers, confirming the association between Gal-1 and decreased survival (HR: 2.12; 95% CI: 1.71–2.64; *p* < 0.001) [69]. In 2018, Long et al. analyzed a total of 2093 GC patients, including eight retrospective case/control studies, and found that the high expression of Gal-1 or low expression of galectins-3, -8, and -9 were significantly linked to poorer prognosis in GC patients [70]. Notably, this study incorporated two investigations on Gal-1 expression in GC. Finally, in 2018, You et al. reviewed 127 cases of GC, identifying Gal-1 expression and the presence of VM as the indicators of poor prognosis [59].

Previous publications have evaluated the clinicopathological characteristics associated with Gal-1 expression in the stroma of GC [47,59,63,64,65,66,67]. Tumors with Gal-1 overexpression have been linked to features such as advanced TNM stage, lymphovascular invasion, or lymph node metastases. Moreover, earlier investigations have revealed associations with tumor depth, size, location, perineural infiltration, or serosal invasion. Although our study did not confirm the relationship between some of these findings and Gal-1 expression, we found a significant correlation between stromal Gal-1 overexpression and various clinicopathological characteristics, including the presence of necrosis, lymphovascular invasion, or advanced pTNM stage. Additionally, epithelial Gal-1 overexpression was significantly related to peritumoral lymphocytic infiltration. The relationship between lymph node involvement, tumor grade, and stromal and epithelial Gal-1 overexpression, respectively, tended to be significant.

The association observed in our study between stromal Gal-1 overexpression and the presence of necrosis may be attributed to the role of Gal-1 in creating a microenvironment conducive to tumor progression. As documented in the prior literature, free Gal-1 promotes tumor angiogenesis and protects the tumor from immune attack by inducing the apoptosis of effector T lymphocytes [72,73]. Moreover, evidence suggests that tumor cell death via necrosis inhibits an adequate immune response, and effector T lymphocytes are not identified in the hypoxia-exposed necrotic tumor areas [74]. Furthermore, these hypoxia-exposed areas are sites at risk for the development of more aggressive and treatment-resistant cell phenotypes [75].

We have also demonstrated an association between the presence of peritumoral lymphocytic infiltration and epithelial Gal-1 overexpression, although, for Gal-1 expression in the stroma, only a trend of association with peritumoral lymphocytic infiltration was identified (*p* = 0.06). However, our study failed to demonstrate a relationship between Gal-1 expression and the presence of intratumoral infiltrates (*p* > 0.05). This finding contradicts the previous reports where Gal-1 was described to create an immune evasion environment, altering cytokine production and triggering a proapoptotic effect on T lymphocytes [76,77,78,79,80]. Additionally, Gal-1 blockade has been reported to induce the positive regulation of CD4+ and CD8+ T-lymphocyte tumor infiltrates in pancreatic adenocarcinoma, head and neck carcinoma, melanoma, neuroblastoma, lung adenocarcinoma, ovarian carcinoma, and breast carcinoma [52,55,78,81,82,83,84]. A recent analysis showed that Gal-1 not only induces the apoptosis of T lymphocytes but can also reprogram regulatory CD8+ T-cells, increasing their immunosuppressive capacity [85]. Regarding B lymphocytes, Gal-1 seems to promote the regulatory immunosuppressive function of certain subsets, such as T2 and T1 B cells [86]. On the other hand, recent studies suggest that Gal-1 may play a proinflammatory role in certain diseases, such as sepsis or specific infections [31]. Characterizing the subsets of lymphocytes in the tumor microenvironment in subsequent studies could clarify the role of Gal-1 in lymphocytic infiltration in GC.

Finally, Gal-1 has potential value as a therapeutic target. As previously discussed, Gal-1 expression in tumor tissue can act as a biological modifier in tumor growth, invasion, angiogenesis, and metastasis, creating a tumor microenvironment that facilitates tumorigenesis. These functions can decisively influence patients’ treatment response. Thus, some reports indicate that Gal-1 induces resistance to specific treatments in certain malignancies, such as kinase inhibitors (sorafenib) in HCC or cisplatin in cervical squamous cell carcinoma and HCC [87,88,89]. Interestingly, Gal-1 also facilitates the action of other compounds, such as vincristine in B-lymphoblastic lymphoma and paclitaxel in ovarian cancer [90,91]. Numerous articles have analyzed Gal-1 inhibition as a therapeutic tool in diverse tumors [22,78,92]. For instance, recent reports demonstrate that Gal-1 blockade significantly increases intratumoral T-cell infiltration, leading to a better response to anti-PD-1 therapy [78]. Specific Gal-1 inhibitor efficacy has been described in oral squamous cell carcinoma, thyroid carcinoma, HCC, ovarian carcinoma, breast carcinoma, or small cell lung carcinoma [21,22,52,87,93,94].

## 5. Strengths and Limitations of the Study

### 5.1. Strengths

This study presents a homogeneous set of findings derived from a group of Western patients from Spain. All the patients were resectable cases diagnosed and treated in a tertiary hospital, and the histological and IHC features were independently reviewed by two pathologists following a detailed histological protocol.

### 5.2. Limitations

This study is retrospective in nature, which inherently introduces potential limitations in terms of data collection, the inference of causality, and susceptibility to biases. GC is less prevalent in Western countries compared to Asian countries, resulting in a smaller pool of patients available for analysis. Our cohort represents a homogeneous Spanish population of resectable patients; therefore, conclusions about other ethnicities, particularly those originating from Asian countries, and about non-surgical cases should not be extrapolated. IHC study was performed on the TMA sections, which do not represent the entirety of the sampled tumor. To overcome this limitation, cores were selected from both the central and peripheral tumor areas, with no significant differences observed in staining between them. Finally, IHC has inherent limitations as a semi-quantitative technique prone to interobserver variability. To mitigate these differences, the Gal-1 IHC was evaluated by two independent pathologists.

## 6. Conclusions

The findings from our study highlight the significance of Gal-1 in tumor progression and prognosis among Western patients with GC. Gal-1, which has been linked to tumor growth and aggressiveness in various tumor types, was frequently expressed in both the stromal and epithelial compartments of GC tissues. Specifically, the high stromal expression of Gal-1 was significantly correlated with aggressive clinicopathological features and poorer CSS in our cohort of Western patients. Moreover, stromal Gal-1 overexpression emerged as an independent prognostic factor for CSS in multivariable analysis. These results underscore the potential of Gal-1 as a prognostic marker and therapeutic target in GC, suggesting the feasibility of developing targeted drugs against this protein to improve patient outcomes. Demonstrating Gal-1 overexpression could become a cost-effective technique for selecting GC patients for targeted therapy in the future. Additionally, combining anti-Gal-1 therapies with immunotherapy in these patients could enhance the effectiveness of immunotherapeutic drugs.

Further investigations into the mechanisms underlying Gal-1-mediated tumor progression and the development of effective Gal-1-targeted therapies may offer promising avenues for the management of GC in Western regions. In addition, expanding the scope of research to encompass larger studies across diverse populations will be crucial to validate our findings.

## Figures and Tables

**Table 1:** Main clinicopathological features of our series.

Features		Patients, n (Valid %), n = 149
Age (years) [median (range)]		76 (33–91)
Male gender		82 (55)
Symptoms	Symptomatic	106 (89.3)
	Local symptoms	76 (65)
	Systemic symptoms	65 (55.1)
Size, mm [median (range)]		50 (10–120)
Depth, mm [median (range)]		10 (1–29)
Macroscopic type	Polypoid	31 (21.8)
	Flat	11 (7.7)
	Ulcerative	45 (31.7)
	Fungoid	55 (38.7)
Location	Cardias	2 (1.5)
	Fundus	12 (9.2)
	Body	43 (33.1)
	Antrum	73 (56.2)
Laurén subtype	Intestinal	87 (59.2)
	Diffuse	43 (29.3)
	Mixed	17 (11.6)
High grade		78 (53.1)
Necrosis		40 (27)
Signet ring cells		63 (42.9)
Lymphovascular invasion		64 (43.2)
Perineural infiltration		67 (45.3)
Advancing front (infiltrative)		91 (61.9)
Budding		24 (26.1)
Desmoplasia		72 (49.7)
Intratumoral lymphocytic infiltration	Mild/moderate	25 (18.1)
	Intense	105 (76.1)
Peritumoral lymphocytic infiltration		40 (27.4)
Lymph node metastases		96 (67.6)
Number of metastatic lymph nodes [median (range)]		3 (1–47)
pT	T1	8 (5.5)
	T2	29 (19.9)
	T3	90 (61.6)
	T4	19 (13)
pN	N0	46 (32.4)
	N1	26 (18.3)
	N2	37 (26.1)
	N3	33 (23.2)
pTNM	I	21 (15.2)
	II	51 (37)
	III	66 (47.8)
Gastrectomy	Subtotal	105 (70.9)
	Total	43 (29.1)
Lymphadenectomy	D1	7 (4.7)
	D2	29 (19.5)
	NS ^a^	113 (75.8)
Number of LN ^b^ resected	≥16	87 (61.3)
	<16	55 (38.7)
Adjuvant therapy		26 (23.2)
Recurrence		64 (44.1)
Death due to tumor		37 (26.6)

^a^ NS: not specified, ^b^ LN: lymph nodes.

**Table 2:** Immunohistochemical features of Gal-1 positive cases.

Epithelial Expression (n = 95)		n	%
Expression pattern	Cytoplasmic	82	86.3
	Nuclear	0	
	Cytoplasmic and nuclear	8	8.4
	Cytoplasmic and membranous	5	5.3
Staining intensity	Mild	69	72.6
	Moderate	26	27.4
**Stromal Expression (n = 143)**		**n**	**%**
Expression pattern	Cytoplasmic	143	
	Nuclear	0	100
Staining intensity	Mild	87	58.4
	Moderate	56	41.6

**Table 3:** Relationship between Gal-1 expression in stroma and clinicopathological variables.

Feature		Gal-1 Neg	Gal-1 +	*p*
Necrosis		19.8%	42.6%	0.004
Lymphovascular invasion		37.6%	55.3%	0.043
pTNM stage (II–III)		79.7%	95.5%	0.017
Recurrence		38.14%	56.2%	0.039
Death due to tumor		15.4%	47.9%	<0.001
*Peritumoral lymphocytic infiltration*		*22.8%*	*37.8%*	*0.060*
*Lymph node metastases*		*62.5%*	*78.3%*	*0.060*
*pN*	*pN0*	*37.5%*	*21.7%*	*0.066*
	*pN1*	*16.7%*	*21.7%*	
	*pN2*	*28.12%*	*21.7%*	
	*pN3*	*17.7%*	*34.8%*	

Variables in italics approached significance but were not statistically significant (*p* > 0.05).

**Table 4:** Relationship between Gal-1 expression in epithelium and clinicopathological variables.

Feature	Gal-1 Neg	Gal-1 +	*p*
Lymphovascular invasion	34.9%	54.8%	0.016
Peritumoral lymphocytic infiltration	21.2%	36.1%	0.047
Death due to tumor	19.5%	35.5%	0.034
*Tumor grade (high)*	*46.5%*	*62.3%*	*0.059*
*pTNM stage (II–III)*	*80.5%*	*91.1%*	*0.089*

Variables in italics approached significance but were not statistically significant (*p* > 0.05).

**Table 5:** Univariable analysis: significant clinicopathological characteristics associated with tumor-related mortality.

Feature		OR (95% CI ^a^)	*p*
Necrosis		2.5 (1.1–5.5)	0.027
Laurén (diffuse)		2.3 (1–5.2)	0.039
pN	pN0	1	0.029
	pN1	1.7 (0.5–5.4)	
	pN2	0.9 (0.3–3)	
	pN3	3.8 (1.3–10.9)	
Stromal Gal-1 +		5.1 (2.3–11.3)	<0.001
Epithelial Gal-1 +		2.3 (1.1–4.9)	0.034
LNR ^b^ (median)		0.25 vs. 0.1	0.011
Metastatic lymph nodes (median)		4 vs. 2	0.043
Age at diagnosis (median)		68 vs. 76	0.013
*Signet ring cells*		*2.15 (1–4.7)*	*0.051*

^a^ CI: confidence interval; ^b^ LNR: lymph node ratio. Variables in italics approached significance but were not statistically significant (*p* > 0.05).

**Table 6:** Multivariable analysis (Cox regression). Independent risk factors for death from gastric cancer.

Covariates	HR (95% CI ^a^)	*p*
Laurén (diffuse)	2.65 (1.26–5.59)	0.011
LNR ^b^	7.71 (2.72–21.86)	<0.001
Stromal Gal-1 +	3.93 (1.84–8.4)	<0.001

^a^ CI: confidence interval; ^b^ LNR: lymph node ratio.

## Data Availability

The original contributions presented in the study are included in the article, further inquiries can be directed to the corresponding authors.
